# The effect of methyl sulphonyl methane supplementation on biomarkers of oxidative stress in sport horses following jumping exercise

**DOI:** 10.1186/1751-0147-50-45

**Published:** 2008-11-07

**Authors:** Gonzalo Marañón, Bárbara Muñoz-Escassi, William Manley, Cruz García, Patricia Cayado, Mercedes Sánchez de la Muela, Begoña Olábarri, Rosa León, Elena Vara

**Affiliations:** 1 Horsepital SL. Madrid, Spain; 2 Departamento de Bioquímica y Biología Molecular. Facultad de Medicina. Universidad Complutense de Madrid. Madrid, Spain; 3 Departamento de Medicina Animal y Cirugía. Facultad de Veterinaria. Universidad Complutense de Madrid. Madrid, Spain

## Abstract

**Background:**

Exercise induces changes in several organs and tissues, and this process might be due to oxidative damage caused by free radicals and inflammatory mediators. Methyl Sulphonyl Methane, better known as MSM, is a naturally occurring sulphur compound with well-known antioxidant properties. On the other hand, Vitamin C is important in limiting free radical damage in the aqueous phase of the cell, and cellular vitamin C status may be linked to the mechanisms involved in quenching cellular reactive oxygen species. The aim of this study was to determine if supplementation with MSM and vitamin C could alleviate exercise-induced oxidative stress in horses undergoing jumping competition.

**Methods:**

Twenty four jumping horses involved in competition were used. Horses were given the following three treatment diets: control (without supplementation), MSM 8 mg/kg, and combined supplements (MSM 8 mg/kg + Vit-C 5 mg/kg). EDTA blood samples were collected before exercise, upon arrived to the schooling area (control), and each week after last show. Nitric oxide, carbon monoxide, lipid hydroperoxides and the antioxidant enzymes, glutathione peroxidase, glutathione transferase and glutathione reductase, plasma levels were determined.

**Results:**

Competition induced a significant increase in lipid peroxidation, nitric oxide and carbon monoxide. By contrary, reduced glutathione as well as antioxidant enzyme activities, were decreased. MSM administration significantly ameliorated all these exercise-related changes, and this effect was potentiated by Vit C reaching values in some of the parameters similar to those found before competition.

**Conclusion:**

These results suggest that jumping exercise could induce harmful effects on horses, probably due to an increase in oxidative damage and proinflammatory molecules. In addition, we have demonstrated that MSM could exert some protective effect on oxidative and inflammatory exercise-induced injury.

## Background

Exercise is accompanied by several changes in the morphology and physiology of different organs and tissues. One of the theories that tries to explain the exercise effects defends that it may be in part due to the accumulation of oxidative damage induced by reactive oxygen species (ROS) and reactive nitrogen species (RNS) to cells and macromolecules [1-3]. ROS are highly reactive molecules which are mainly generated in mitochondria during oxygen metabolism [4]. Approximately 95% of the oxygen consumed is reduced to water during aerobic metabolism, but the remaining fraction may be converted to reactive oxygen species and other free radicals, inducing oxidative stress. There may be a number of sources of this oxidative stress, including mitochondrial superoxide production, ischemia-reperfusion mechanisms and auto-oxidation of catecholamines. Severe or prolonged exercise can overwhelm antioxidant defences, which include vitamins E and C and thiol antioxidants, which are interlinked in an antioxidant network, as well as antioxidant enzymes. Evidence for oxidative stress and damage during exercise comes from direct measurement of free radicals, from measurement of damage to lipids and DNA, and from measurement of antioxidant redox status, especially glutathione. There is little evidence that antioxidant supplementation can improve performance, but a large body of work suggests that bolstering antioxidant defences may ameliorate exercise-induced damage.

On the other hand, strenuous and/or damaging exercise elicits a stress response analogous to the acute-phase immune response. Exercise-induced tissue damage and/or increased reactive oxygen species production stimulate cytokine production, up regulating the inflammatory cascade [5]. Two molecules that have been recently involved in oxidative damage and inflammatory response are Nitric oxide (NO) and Carbon monoxide (CO). Nitric oxide can act as both inflammatory mediator and RNS, either directly or through peroxynitrites generated by its interaction with O_2_^- ^[6,7]. CO is one of the elements of the Hemo-oxygenase 1-CO pathway, which has been proposed to constitute a defence system against oxidative and inflammatory damage [8]. Both NO and CO activate soluble-guanilyl cyclase (sGC), thus inducing and increase in intracellular cyclic-guanilyl monophosphate (cGMP) as second messenger [8,9].

Vitamin C (ascorbic acid) is a powerful natural antioxidants in mammals [10]. It is present in high concentrations in leukocytes and there is evidence that it is involved quenching cellular reactive oxygen species. However, results from previous studies examining the effects of supplementation with Vitamin C exercise-stimulated oxidative damage have been inconclusive: inhibition of lipid peroxidation [11], no effect [12], and even increased lipid peroxidation [13]. Possible reasons for these inconsistencies include differences in modes, duration, and intensity of exercise.

On the other hand, Methyl Sulphonyl Methane, better known as MSM, is a naturally occurring sulphur compound that MSM may plays a role in the synthesis of glutathione (GSH) [14] one of the most important intracellular antioxidants [15], through its transulfuration pathway [14].

MSM has demonstrated to exert protective effects against many diseases in humans, such as hyperacidity, parasites, constipation, musculoskeletal pain, arthritis, allergies and immunomodulation [16-18]. However, to our knowledge little is known about the mechanism by which MSM may exert its protective effect against exercise-induced oxidative stress in horses.

The purpose of the present study was to determine whether exercise-induced oxidative stress responses could be alleviated by supplementation with MSM and vitamin C in horses undergoing jumping competition.

For this purpose, NO, CO lipid hydroperoxides (LPO), glutathione plasma levels were determined after intensive jumping competition in horses. Additionally, we also determined plasma antioxidant enzyme (glutathione peroxidase, glutathione transferase and glutathione reductase) activities.

## Methods

Twenty for jumping horses (8–13 years old; 2 stallions, 13 geldings, 9 mares) competing in the south of Spain (Winter Sunshine Tour) during 5 weeks (3–4 days/week), were used in the present study. All horses were apparently healthy and showed a good performance condition, Horses were randomly assigned to one of three experimental groups: MSM group (8 mg/Kg of MSM, Alefa Aesar, Germany: 98–99% purity, daily); MSM+Vit C group (8 mg/Kg of MSM plus 5 mg/Kg of vitamin C, daily) and Control (no supplementation). Supplements (one daily oral dose mixed in the morning feed), were administered in spaced doses, 7 days before arrived to the schooling area and every day until the end.

Weight average (540 ± 47 Kg), estimated by standardized measuring tape around the thorax, and was similar in all groups.

Blood samples were collected from the jugular vein of each horse. The first sample was collected one day after arrival to the show ground (in the morning, 90 minutes after feeding and before exercise). All the other samples were collected 10–15 minutes after competition, at weekly intervals (all horses were warmed up for 20–30 minutes and then jumped over a course of 500 meters with 16 jumps at an average speed of approximately 350–400 meters/minute).

Blood was drawn into 5 cc purple-top Vacutainer tubes (containing 1 mg/mL EDTA). Blood was centrifuged in a standard centrifuge (Labofuge 300, Heraeus Holding, Germany) at 3000 g × 10 min; plasma was then aliquoted for various assays and stored at -80°C until day of analysis.

The research project was approved by our institution (Universidad Complutense de Madrid) and by the "Real Federación Hípica Española". Prior to initiation the study, an informed consent was provided by horse's riders, as well as by the owners.

For GSH assessment, a specific colorimetric method was used [19]. Briefly, glutathione was sequentially oxidized by 5-5' dithio-bis (2-dinitrobenzoic acid) (DTNB) and reduced by NADPH in the presence of glutathione disulfide reductase, which results in the formation of 5-thio-2-nitrobenzoic acid (TNB). The rate of TNB formation is measured spectrophotometrically at 412 nm.

Plasma levels of LPO and GPx, GST and GR activities were measured by commercially available kits (Cayman Chemical Company, Ann Arbor, USA).

NO plasma concentration was measured by the Griess reaction as NO_2 _concentration after NO_3 _reduction to NO_2 _as currently performed in our laboratory [20]. Briefly, samples were deproteinized by the addition of sulfosalicylic acid, were then incubated for 30 min at 4°C, and subsequently centrifuged for 20 minutes at 12,000 g. After incubation of the supernatants with Escherichia coli NO_3 _reductase (37°, 30 min), 1 ml of Griess reagent (0.5% naphthylenediamine dihydrochloride, 5% sulfonilamide, 25% H_3_PO_4_) was added. The reaction was performed at 22 °C for 20 min, and the absorbance at 546 nm was measured, using NaNO_2 _solution as standard. The measured signal is linear from 1 to 150 μM (r = 0.994, P < 0.001, n = 5), and the detection threshold is ~2 μM.

To quantify the amount of CO, the ratio of carboxy-haemoglobin after haemoglobin addition was measured [21]. Haemoglobin (4 μM) was added to samples and the mixture was allowed to react for 1 min, to be sure of a maximum binding of CO to haemoglobin. Then, samples were diluted with a solution containing phosphate buffer (0.01 mol/L monobasic potassium phosphate/dibasic potassium phosphate, pH 6.85) containing sodium dithionite, and after 10 min at room temperature, absorbance was measured at 420 and 432 nm against a matched curve containing only buffer [21].

Reproducibility within the assays was evaluated in three independent experiments. Each assay was carried out with three replicates. The overall intra-assay coefficient of variation has been calculated to be <5%. Assay to assay reproducibility was evaluated in three independent experiments. The overall inter-assay coefficient of variation has been calculated to be <6%.

### Statistical analysis

Results are expressed as the mean ± SEM, from n = 8. Mean comparison was done by the Kuskal-Wallis test followed by a Mann Whitney test; a confidence level of 95% (p < 0.05) was considered significant.

## Results

**Figure 1:**
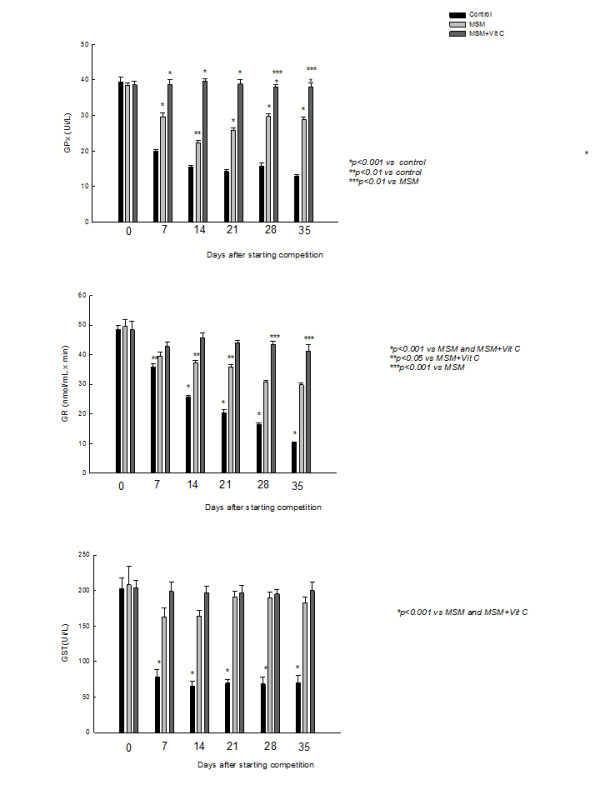
**Effect of methyl sulphonyl methane (MSM), alone or combined with vitamin C (Vit C), on training-induced decrease of glutathione peroxidase (GPx), glutathione transferase (GST) and glutathione reductase (GR) activities.** Values are expressed as mean ± SEM.

As shown in figure 1, exercise decreased all antioxidant enzymes studied. GR activity was reduced by exercise in a time-dependent manner, while no changes were observed in GPx and GST activities during time. These effects were partially prevented by MSM, although levels remain lower than those observed on week 0, before starting competition. When MSM was combined with Vit C, the three enzymatic activities were restored reaching normal values (before starting competition).

**Figure 2:**
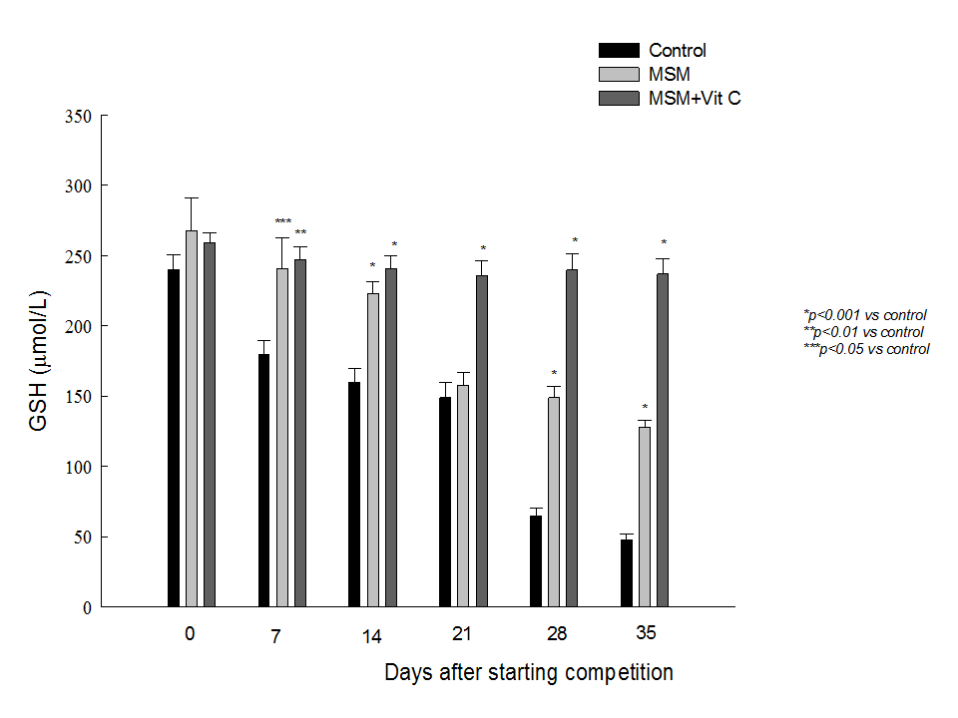
**Effect of methyl sulphonyl methane (MSM), alone or combined with vitamin C (Vit C), on training -induced decrease of reduced glutathione (GSH) plasma levels.** Values are expressed as mean ± SEM.

Exercise resulted in a dramatic decrease in GSH plasma levels (Fig. 2), while mean plasma oxidized glutathione (GSSG) did not significantly change suggesting that GSH may play a central antioxidant role in plasma during intensive physical exercise and that its modifications are closely related to exercise intensity. MSM induced an increase in GSH levels, and again the MSM effect was potentiated by Vit C, reaching values comparable to those observed before exercise.

**Figure 3:**
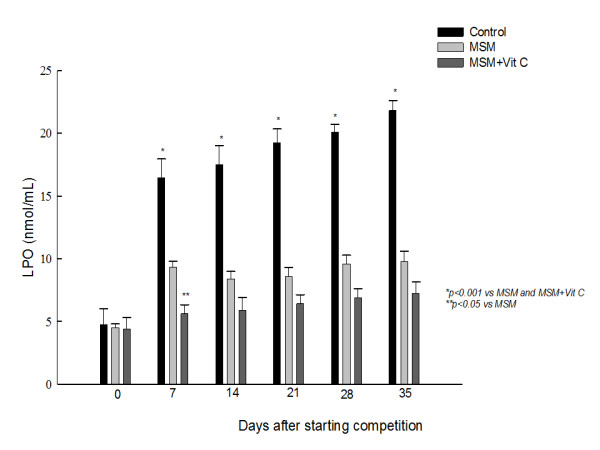
**Effect of methyl sulphonyl methane (MSM), alone or combined with vitamin C (Vit C), on training -induced increase of lipid hydroperoxides (LPO) levels.** Values are expressed as mean ± SEM.

LPO content was higher after exercise as compared to those before exercise, (Fig 3). When animals were supplemented with MSM or MSM+Vit C, a reduction in LPO content was observed.

**Figure 4:**
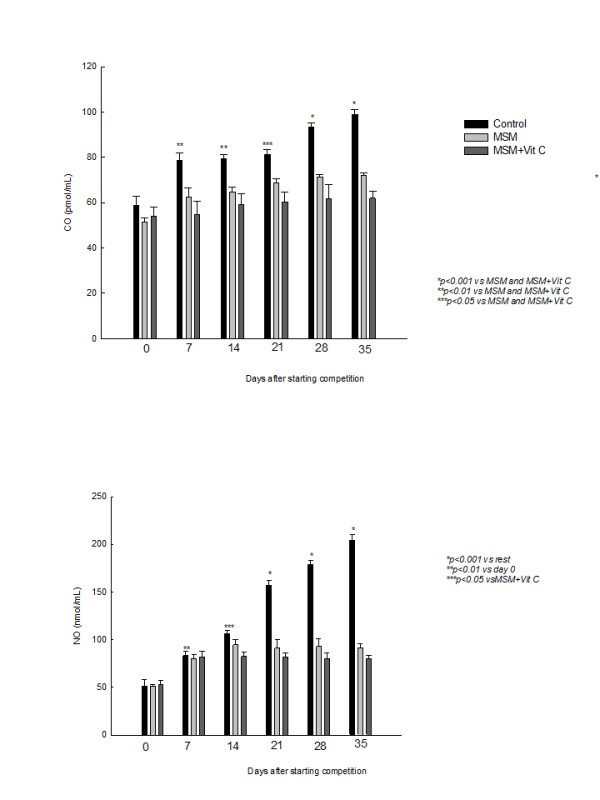
**Effect of methyl sulphonyl methane (MSM), alone or combined with vitamin C (Vit C), on training -induced changes on carbon monoxide (CO) and nitric oxide (NO) plasma levels.** Values are expressed as mean ± SEM.

As shown in the figure 4A, exercise also induced an increase in CO plasma levels of horses after exercise. When MSM, alone or in combination with Vit C, was administered, a reduction in CO release was found.

NO plasma level was increased with exercise (Fig 4B). When horses were supplemented with MSM, no significant effect was observed during the first days, but it reduced the increase in NO levels after intensive exercise (several weeks competing).

## Discussion

The role of ROS in the exercise-induced damage is supported by many studies. ROS production has been found to increase with exercise, thus augmenting the amount of oxidative damage induced to lipids, proteins and DNA [1,2,22,23]. The control of ROS is important to the athlete because they interfere with the rebuilding process which is so necessary after strenuous competition. Therefore, many studies have focused their attention on the search of substances that could restrain this increase in exercise-induced oxidative stress.

Vitamin C and MSM are known antioxidants which can scavenge ROS, thus preventing tissue damage. MSM, an endogenous cellular metabolite that acts as sulphur donor in many transmethylation reactions, is also able to act as an antioxidant and free radical scavenger. It has also shown to exert protective effects on different experimental pathological situations, in which free radicals and ROS are involved, such as hyperacidity, parasites, constipation, musculoskeletal pain, arthritis, allergies, Ehlers-Dantos syndrome and immunomodulation [16,17,24]. Thus, it seemed interesting to look into the possible protective effect of this molecule on the effects of strenuous exercise, in which free radicals seem to be involved.

A decrease in GSH-related enzyme activities after exercise has been found in this study. This finding is in accordance with previous studies, in which a decrease of these activities was observed [25-27]. However, to our knowledge this is the first work showing a decrease in GSH-related enzymes after jumping competition. This reduction in GPx and GST activities would lead to a decrease in GSH synthesis, which would affect many essential metabolic pathways in which GSH is involved.

The glutathione (GSH) antioxidant system is foremost among the cellular protective mechanisms. Depletion of this small molecule is a common consequence of increased formation of reactive oxygen species during increased cellular activities. This phenomenon may occur in the lymphocytes during the development of an immune response or in muscular cells during strenuous exercise. Oxidative stress could be involved in this exercise-related decrease in enzyme activities. This situation would lead to a self-perpetuating cycle, in which the free radicals generated would induce GSH depletion, thus increasing oxidative stress that would reduce antioxidant enzyme levels, which would further reduce GSH synthesis. Exhaustive exercise depletes glutathione and simultaneously generates free radicals. This is evidenced by increases in lipid peroxidation, glutathione oxidation, and oxidative protein damage [28]. In our study exercise induced an increase in cellular oxidative stress (as shown by the increase in LPO content) and NO levels, and these factors could account for the increase in oxidative stress.

A reduction in GSH levels was also found, which could be both, cause or consequence of the decrease in GPx and GR activities. Supplementation to the horses with MSM was able to provoke a recovery of GPx and GR activities, presumably due to a reduction in oxidative and inflammatory damage induced by the improvement of GSH, and NO levels, as will be discussed later.

GSH is the most important intracellular antioxidant thiol, and the liver is its main source [15]. It has been shown that exercise induces perturbations in blood glutathione redox status [22,29-31] and these reports are in accordance with our present findings. This fact could be either cause or consequence of the increase in oxidative damage found after exercise. In our study, supplementation with MSM induced an increase in GSH levels, as could be expected, since MSM metabolism provides one of the precursors needed for GSH synthesis, therefore counteracting GSH depletion.

As it has been previously mentioned, free radicals have been implicated as mediators on exercise-induced cellular dysfunction and cell to cell signalling. On the other hand, it is known that mitochondrial production of oxygen-derived radicals is increased with exercise [4], and this fact could be a major mechanism for the exercise-related increase in oxidative stress. Our finding of increased horse's plasma LPO content after jumping exercise supports the hypothesis of augmented oxidative stress with exercise. The exercise-induced increase in LPO content was reverted by supplementation with MSM, pointing to an improvement in the oxidative status of the cells, according to the MSM-induced increase of GSH previously mentioned. Moreover, MSM has been suggested to act as a direct free radical scavenger, a mechanism that could also be involved in its antioxidant properties [14].

Vitamin C is an important water soluble vitamin. As an antioxidant, vitamin C's primary role is to neutralize free radicals. It is in a unique position to scavenge aqueous peroxyl radicals before these destructive substances have a chance to damage the lipids [32]. It works along with vitamin E, a fat-soluble antioxidant, and the enzyme glutathione peroxidase in order to stop free radical chain reactions. Once vitamin C is depleted, uric acid, albumin bound bilirubin and protein thiols only partially reduce lipid peroxidation [32]. The effect of Vitamin C on exercise-stimulated oxidative damage remains controversial [11-13]. In this study we found that the protective effect of MSM against exercise-induced oxidative stress was enhanced by vitamin C. Although our data do not provide a clear explanation about the mechanisms by which vitamin C potentiated the effect of MSM, they suggest that MSM, and vitamin C have common targets or work synergistically to protect cells from oxidative damage.

Some exercise-related pathologies are now considered chronic inflammatory processes. In fact, proinflammatory molecules have been reported to increase with strenuous exercise [33]. Nitric Oxide (NO) is one molecular mediator involved in both the inflammatory response [34] and oxidative damage [6,35]. In the present study, NO plasma levels were found to be increased after exercise, in accordance with other reports, which show that iNOS activity and NO release are increased with exercise in several tissues [36,37]. Our study shows that supplementation with MSM did not modify the increase in NO plasma levels observed after the first day of competition, but is able to reduce the increase in NO levels after intensive exercise (several days competing), suggesting that this molecule may be able to modulate the inflammatory response.

CO is a physiologically synthesized molecule that shares some of the mechanisms of action and physiological effects of NO [8,9]. The main endogenous source of CO is heme metabolism by heme-oxygenase (HO) [8,9]. This HO-CO pathway has been recently proposed to be involved in the defence against oxidative stress and the deleterious effects of NO, since it removes the cytotoxic free heme, and produces some molecules with antioxidant and anti-inflammatory effects, such as biliverdin and CO [8,38-40]. Therefore, this pathway could be activated to counteract an excess of oxidant and inflammatory agents [8]. The present study shows that exercise induces an increase in plasma CO levels, and this could mean that this defence mechanism has been activated by the increase in exercise-associated ROS and proinflammatory molecules, such as NO. We also demonstrate that MSM is able to reduce CO release, and this could be due to the fact that horses supplemented with MSM are exposed to a lesser amount of ROS and RNS, therefore leading to a lower HO induction.

## Conclusion

These results suggest that jumping exercise could induce harmful effects on horses, probably due to an increase in oxidative damage and proinflammatory molecules. In addition, we have demonstrated that MSM could exert some protective effect on oxidative and inflammatory exercise-induced injury. All these findings suggest the necessity of investigating the mechanisms of the protective effect of MSM, in order to develop strategies capable to increase performance in sport horses.

## Abbreviations

MSM: methyl sulphonyl methane; Vit C: vitamin C; GPx: glutathione peroxidise; GST: glutathione transferase; GR: glutathione reductase; HO: heme oxygenase; ROS: reactive oxygen species; RNS: reactive nitrogen species; NO: nitric oxide; CO: carbon monoxide; GSH: glutathione.

## Competing interests

The authors declare that they have no competing interests.

## Authors' contributions

GM: Participated in the design of the study and performed the statistical analysis. BME, WM, MSM, PC, BO, RL and CG: Participated in the recovery of blood samples and carried out the biochemistry determinations. EV: Conceived the study, participated in its design and coordination and helped to draft the manuscript. All authors read and approved the final manuscript.
